# Neoadjuvant checkpoint blockade in combination with Chemotherapy in patients with tripe-negative breast cancer: exploratory analysis of real-world, multicenter data

**DOI:** 10.1186/s12885-023-10515-z

**Published:** 2023-01-07

**Authors:** Heran Deng, Liying Wang, Na Wang, Kejin Zhang, Yanxia Zhao, Pengfei Qiu, Xiaowei Qi, Danhua Zhang, Fei Xu, Jieqiong Liu

**Affiliations:** 1 Guangdong Provincial Key Laboratory of Malignant Tumor Epigenetics and Gene Regulation, Breast Tumor Center, Sun Yat-Sen Memorial Hospital, Sun Yat-Sen University, Yanjiang West Road 107#, Guangzhou, 510120 Guangdong China; 2 State Key Laboratory of Oncology in South China, Collaborative Innovation Center for Cancer Medicine, Sun Yat-Sen University Cancer Center, Sun Yat-Sen University, Dongfeng Road 651#, Guangzhou, 510060 Guangdong China; 3 Xiangya Hospital of Central South University, Center South University, Changsha, Hunan China; 4 Union Hospital Tongji Medical College Huazhong University of Science and Technology, Huazhong University of Science and Technology, Wuhan, Hubei China; 5 Shandong Tumor Hospital, Shandong university, Jinan, Shandong China; 6 The Southwest Hospital of AMU, Army Medical University, Chongqing, Sichuan China; 7 The Second Xiangya Hospital of Central South University, Center South University, Changsha, Hunan China

**Keywords:** Tripe-negative breast cancer, Neoadjuvant immunotherapy, Anti-PD-1/L1 antibody, Real-world study

## Abstract

**Purpose:**

Despite the poor prognosis of triple-negative breast cancer (TNBC), it has been demonstrated that neoadjuvant immunotherapy in combination with chemotherapy can improve the pathologic complete response (pCR) rate and/or long-term outcome of TNBC. However, there have been no real-world studies reporting on the effectiveness of neoadjuvant checkpoint inhibitors in early TNBC.

**Methods:**

Between November 2019 and December 2021, 63 early TNBC patients treated with anti-PD-1 antibodies (pembrolizumab or camrelizumab) or anti-PD-L1 antibody (atezolizumab) in combination with chemotherapy at seven institutions were included. PCR1 defined as ypT0/Tis and ypN0 was the primary endpoint. Secondary endpoints included pCR2 defined as ypT0/Tis, overall response rate (ORR), disease-free survival (DFS), drug-related adverse events (AEs) and biomarkers.

**Results:**

Among the patients in the current study, 34.9% of patients were able to achieve pCR1, and 47.6% of patients had achieved pCR2. The ORR was 82.5%. 33 patients with non-pCR2 tumors were found to have a median DFS of 20.7 months (95% CI 16.3 months-not reached). The DFS of patients with pCR2 and non-pCR2 after neoadjuvant therapy was significantly different (HR = 0.28, 95% CI 0.10–0.79; *P* = 0.038). The most common AEs were nausea (63.4%), fatigue (42.7%), leucopenia (30.0%) and elevated transaminase (11.7%).

**Conclusion:**

It is possible to achieve a meaningful pCR rate and DFS by combining neoadjuvant checkpoint blockade with chemotherapy in patients with high-risk TNBC. Compared to clinical trials, however, there was a slightly lower pCR rate in this multicentered real-world study.

**Supplementary Information:**

The online version contains supplementary material available at 10.1186/s12885-023-10515-z.

## Introduction

The diagnosis of triple-negative breast cancers (TNBCs) refers to those lacking both estrogen receptors (ER and PR) as well as the human epidermal growth factor receptor 2 (HER2). There is a poorer prognosis for TNBC patients compared to those with other subtypes of breast cancer, since it exhibits aggressive clinical behavior and lacks adequate molecular targets for therapy [1]. Previous studies have shown that the pathological complete response (pCR) rate for TNBC after neoadjuvant chemotherapy (NAC) has typically been higher than for breast cancer of other molecular subtypes, and there is a marked reduction in recurrences and deaths among patients who achieve a pCR compared with those who have residual lesions after neoadjuvant therapies [2]. Patients with TNBC, however, approximately 40%-50% achieved a complete response after NAC, and their subsequent treatment selections are limited. Indeed, TNBC is a more immunogenic molecular subtype, containing higher levels of stromal tumor-infiltrating lymphocytes (TILs) and programmed cell death ligand 1 (PD-L1) expression, which suggests a greater immunogenic potential [3–5]. Therefore, patients with TNBC may benefit more from immune-checkpoint inhibitors (ICIs) [6].

In routine clinical practice, chemotherapy combined with PD-1 blockade are commonly given to advanced TNBC patients with PD-L1-positive tumors, because of the positive findings from large phase 3 randomized controlled trials [7–10]. However, the results of trials focusing on neoadjuvant ICI plus chemotherapy in patients with TNBC seemed slightly inconstant. The I-SPY 2 trial, one of the first to assess neoadjuvant immunotherapy, substantially demonstrated an approximately threefold increase in pCR rates in TNBC patients (22% in patients without pembrolizumab versus 60% in patients with pembrolizumab) [11]. In addition, the KEYNOTE-522 trial showed that adding pembrolizumab was able to improve the pCR rates compared with placebo, improving from 51.2% to 64.8% (*P* < 0.001). At its fourth interim analysis, there was a 15.7% relapse rate among patients receiving pembrolizumab in comparison to 23.8% in the placebo group (HR = 0.63, 95% CI 0.48–0.82, *P* = 0.00031), indicating that adding ICI to NAC can also improve the event-free survival [12]. Interestingly, according to the GeparNuevo trial, durvalumab short-term treatment before chemotherapy significantly improved the pCR rate compared to the placebo group (61% vs. 41%, OR = 2.22, 95% CI 1.06–4.64, *P* = 0.035) [13]. However, the results from the NeoTRIPaPDL1 study showed no significant improvement in pCR rates when atezolizumab was added to neoadjuvant therapy with carboplatin and albumin-paclitaxel compared with NAC alone in nonmetastatic TNBC patients [14]. In contrast, according to IMpassion031 findings, patients with TNBC who were treated with NAC combined with atezolizumab achieved a higher pCR rate than those without atezolizumab (41% *vs* 58%; *P* = 0.0044) [15]. Based on the findings of these previous trials, the 2021 NCCN guidelines propose preoperative pembrolizumab combined with carboplatin plus paclitaxel, followed by preoperative pembrolizumab + cyclophosphamide + doxorubicin or epirubicin, followed by pembrolizumab as the first-line neoadjuvant therapy in high-risk patients with TNBC [16].

The results of some trials suggest that neoadjuvant immunotherapy may be effective in treating TNBC. However, there is conflicting evidence, and not all patients can benefit from adding immunotherapy to NAC [17]. Moreover, immunotherapy is accompanied by some immune-related adverse events expected to be irreversible and even life-long damage [18]. Thus, routine clinical practice does not often use neoadjuvant ICI plus chemotherapy. No real-world studies focusing on neoadjuvant immunotherapy in TNBC have been reported so far.

Additionally, neoadjuvant immunotherapy may prove to be an effective and safe treatment for patients with TNBC based on real-world data. To maximize the benefits of immunotherapy-based neoadjuvant treatment, it is imperative that biomarkers be identified to predict efficacy and to select patients better suited for this treatment. Currently, the immune checkpoint inhibitor (ICI) response can be predicted by tumor mutational burden (TMB), commonly defined as the number of non-synonymous mutations in the tumor [19]. However, the prognostic significance of TMB in TNBC patients who underwent neoadjuvant immunotherapy is virtually unknown. To our knowledge, this is the first study to investigate the impact of neoadjuvant immunotherapy combined with chemotherapeutic agents in the real world, which assesses the safety and efficacy and potential biomarkers, including TMB, PD-L1 expression, and BRCA mutation status.

## Patients and methods

### Patient eligibility and study design

This study was a multicenter, retrospective, real-world study (RWS) that included early TNBC patients treated with neoadjuvant immunotherapy between November 2019 and December 2021 across 7 institutions, including Sun Yat-sen Memorial Hospital, Xiangya Hospital of Central South University, Sun Yat-sen University Cancer Center, the Second Xiangya Hospital of Central South University, the Southwest Hospital of AMU, Union Hospital Tongji Medical College Huazhong University of Science and Technology, and Shandong Tumor Hospital. There were several main eligibility criteria for this study: (i) female aged 18–70 years with newly diagnosed, previously untreated nonmetastatic TNBC (HER2/Neu-negative was characterized as immunohistochemistry (IHC) 0–1 + , ER/PR-negative as an ER/PR stain of less than one percent, and HER2/Neu-negative by chromogenic/fluorescent in situ hybridization (FISH) with imaging or biopsy-proven primary breast cancer exceeding 2 cm and/or positive axillary lymph nodes; (ii) complete baseline data and imaging results of at least one examination after treatment as defined by the RECIST v1.1 (Response Evaluation Criteria in Solid Tumors guidelines version 1.1); (iii) with an Eastern Cooperative Oncology Group (ECOG) performance status of 0–2; and (iv) adequate hematologic, hepatic, and renal functions. The critical exclusion criteria included any history of autoimmune disease, infection, or recent use of systemic glucocorticoid, immunostimulants or immunosuppressants, inflammatory breast cancer, allergies, or contraindication to any interventional drugs.

Patients received 200 mg pembrolizumab (*n* = 4) or 200 mg camrelizumab (*n* = 58) once daily, in a 21-day cycle, or 840 mg atezolizumab (*n* = 1) every two weeks in combination with neoadjuvant chemotherapy. Medication details of neoadjuvant chemotherapies are listed in Table 1. The study was reviewed and approved by the Research Ethics Committee of Sun Yat-sen Memorial Hospital, Sun Yat-sen University Cancer Center, Xiangya Hospital of Central South University, the Second Xiangya Hospital of Central South University, the Southwest Hospital of AMU, Union Hospital Tongji Medical College Huazhong University of Science and Technology, and Shandong Tumor Hospital.

**Table 1:** Characteristics of baseline

Characteristics	Number of cases No. (%)
**Age (years)**	
< 35	7 (11.1%)
≥ 35	56 (88.9%)
Median (range)	43 (24–68)
**Menopausal status**	
Postmenopausal	28 (46.6%)
Premenopausal	32 (53.3%)
**Clinical stage before neoadjuvant therapy**	
II	45 (71.4%)
III	18 (28.6%)
**T stage before neoadjuvant therapy**	
T1/T2	37 (58.7%)
T3/T4	26 (41.3%)
**N stage before neoadjuvant therapy**	
N0	18 (28.6%)
N1-3	45 (71.4%)
**Chemotherapy regimen /median duration (weeks)**	
Anthracycline and paclitaxel-based	30 (47.6%); 24.0
Paclitaxel plus carboplatin	23 (36.5%); 21.0
Other	10 (15.9%); 15.0
**Treatment circle**	
8	38 (60.3%)
6	6 (9.5%)
≤ 5	19 (30.2%)
**Checkpoint inhibitor type**	
Anti-PD-1 inhibitor	62 (98.4%)
Anti-PD-L1 inhibitor	1 (1.6%)
**Surgery type**	
Mastectomy	44 (69.8%)
Breast-conserving surgery	19 (30.2%)
**PD-L1 status (CPS score)**	
Positive (≥ 1)	21 (33.3%)
Negative (< 1)	15 (23.8%)
Unknown	27 (42.9%)

### Assessment of tumor mutational burden (TMB)

In this study, of 63 samples of primary breast cancer collected before immunotherapy, 16 received next-generation sequencing (NGS) analysis (OncoScreen Plus, detecting 520 genes closely related to cancer mechanism, and conducted targeted therapy at Burning Rock Dx-Guangzhou Institute (http://www.brbiotech.com); or FoundationOne CDx (F1CDx), which is composed of 324 genes customized by FoundationOne-China Institute California, USA (http://www.foundationmedicine.com); Genecast, involving 306 genes, were tested for genetic variation in Wuxi, Jiangsu Province laboratory (https://www.genecast.com.cn)). Tumor mutational burden (TMB) was determined by analyzing somatic mutations, including substitution of bases and fragment insertion and deletion. The classification of TMB-high (TMB ≥ 5 mut/Mb) and TMB-low was described in prior studies [20].

### Endpoints and assessments

The primary endpoint of this study was the complete pathological response (pCR) rate defined as ypT0/Tis and ypN0 (pCR1). Secondary endpoints included pCR2 defined as ypT0/Tis, overall response rate (ORR), disease-free survival (DFS) as assessed by clinical examination and imaging according to RECIST v1.1and safety. DFS was measured as the time from surgery until disease recurrence or death from any cause.

Pathologists evaluated the tissues obtained by surgery after neoadjuvant therapy. According to RECIST v1.1, the degree of disease extent was assessed by clinical examination and imaging (ultrasound, mammography, and MRI). Patients exhibiting stable disease (SD), or progressive disease (PD) were considered non-responders. Estimated ORR is based on the percentage of complete response (CR) and partial response (PR). Patients without the observed event or failed follow-up were reviewed at the last appropriate visit date. Reporting and grading of adverse events (AEs) was performed using the National Cancer Institute Common Terminology Criteria for Adverse Events (NCI-CTCAEs) version 5.0.

### Statistical analyses

Comparing categorical variables among patient groups was performed using Pearson’s χ^2^ test or Fisher’s exact test. Nonparametric data were analyzed using Mann–Whitney U tests. Median survival time was estimated using Kaplan‒Meier curves with 95% confidence intervals (CI), with significance determined by the log-rank test (two-sided *P* < 0.05). Based on univariable Cox proportional hazard models, hazard ratios (HRs) were calculated to estimate relative risks. SPSS (IBM Corporation, Version 26.0, USA), R (version 3.6.3) (statistical analysis and visualization), R package (ggplot2) (version 3.3.3) (for visualization), survival package R (version 0.4.9) (for visualization) and survival package R (version 3.2–10) (for statistical analysis of survival data) were used for statistical analyses.

## Results

### Characteristics of baseline

From March 2020 to December 2021, a total of 63 early or locally advanced TNBC patients who received neoadjuvant anti-PD-1/L1 inhibitors plus chemotherapy were included. An overview of baseline characteristics is presented in Table 1. Most patients (88.9%) were aged ≥ 35 years old, and a median age of 43 years was at the time of breast cancer diagnosis (range 24–68 years). Premenopausal (53.3%) women accounted for the majority, which was consistent with the high incidence rate of premenopausal breast cancer cases in China. Patients with stage II disease accounted for 71.4%, while those with stage III disease accounted for 28.6%. The ECOG status was 0–1 in all patients, which is not specifically listed in Table 1. 35 (58.7%) patients had tumors smaller than 5 cm, and 25 (41.3%) patients had tumors greater than 5 cm, and 45 (71.4%) patients had positive nodes at diagnosis, while only 18 (28.6%) patients had negative nodes at diagnosis. Half of the included patients underwent anthracycline in combination with paclitaxel-based chemotherapy regimen, which was the NCCN guidelines recommended neoadjuvant chemo-regimen, whereas approximately 36.5% of patients were treated with carboplatin-based chemotherapy, and five cases (8.3%) were treated with eribulin and/or apatinib. Most of the patients (60.3%) completed 8 cycles of neoadjuvant therapy. In terms of types of checkpoint blockade, camrelizumab was administered to 92.1% of patients, and pembrolizumab was administered to 6.3% of patients, while anti-PD-L1 antibody (atezolizumab) was used to just one (1.6%) patient. Among all patients, 30.2% underwent breast-conserving surgery, and 69.8% received a mastectomy. Half of the pathological specimens of the included patients were available for tumor PD-L1 testing, of which 33.3% were PD-L1 positive (regarded as CPS ≥ 1 through 22C3 assay).

### Efficacy

The numbers of patients who achieved a pCR1 (ypT0/Tis and ypN0) or pCR2 (ypT0/Tis) were 22 (34.9%) and 30 (46.7%), respectively. The ORR in this study was 82.5%.

**Fig. 1:**
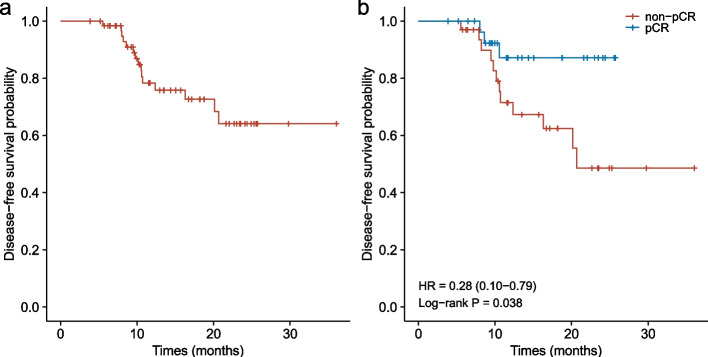
Kaplan–Meier plot for DFS in patients treated with neoadjuvant immunotherapy. **a** Kaplan–Meier plot for DFS in all patients (*n* = 63). **b** Kaplan–Meier plot for DFS in patients with pCR2 (*n* = 30) or non-pCR2 (*n* = 33). pCR2 defined as ypT0/Tis. DFS, disease-free survival. CI, confidence interval. HR, hazard ratio. pCR, complete pathological response rate

There were too few DFS events in all 63 patients to calculate the median DFS (Fig. 1a). The median DFS of 33 patients with non-pCR defined as ypT0/Tis (pCR2) was 20.7 months (95% CI 16.3 months-not reached) (Fig. 1b). In patients who achieved a pCR2 after neoadjuvant therapy, DFS was significantly different from that of patients with non-pCR2 tumors (HR = 0.28, 95% CI 0.10–0.79; *P* = 0.038) (Fig. 1b). And the 1-year DFS was 87.2% (95% CI, 74.5%-100%) in patients achieving pCR2, and 67.3% (95% CI, 51.7%-87.6%) in those with non-pCR2 tumors, respectively; the 2-year DFS was 87.2% (95% CI, 74.5%-100%) and 48.6% (95% CI, 30.6%-77.0%), respectively. However, in patients who achieved a pCR1, DFS was not statistically different from that of patients with non-pCR1 tumors (HR = 0.50, 95% CI 0.17–1.47; *P* = 0.274) (Supplementary Fig. 1). And the 1-year DFS was 83.1% (95% CI, 67.0%-100%) in patients achieving pCR1 and 72.8% (95%CI, 58.9%-89.9%) in those with non-pCR1 tumors, respectively; the 2-year DFS was 83.1% (95% CI, 67.0%-100%) and 57.1% (95% CI, 40.2%-81.0%), respectively.

We also conducted a subgroup analysis to study the outcomes of the patients treated with anthracycline and taxane-based chemotherapy (with or without carboplatin) (*n* = 30). We found that the pCR1 and pCR2 was 33.3% (10/30) and 43.3% (13/30), respectively. And there were too few DFS events in each subgroup to calculate the median DFS (Supplementary Fig. 2a). In patients who achieved a pCR1, DFS was not statistically different from that of patients with non-pCR1 tumors (HR = 0.37, 95% CI 0.07–2.00, *P* = 0.349) (Supplementary Fig. 2b). Also, in patients who achieved a pCR2 after neoadjuvant therapy, DFS was not significantly different from that of patients with non-pCR2 tumors (HR = 0.23, 95% CI 0.05–1.12; *P* = 0.136) (Supplementary Fig. 2c).

The results for univariate adjusted composite outcome HRs for recurrence risk factors are displayed in Table 2. Based on the results of the univariate analysis, we found that DFS did not differ significantly regarding age, menopausal status, baseline T, N or clinical stage, chemotherapy regimen, treatment cycle, PD-L1 status or BRCA mutation status.

**Table 2:** Univariate of factors predicting disease-free survival

Characteristics	Univariable cox	
	**HR (95% CI)**	***P*-value**
**Age**	0.567 (0.159–2.028)	0.383
**Menopausal status**	1.760 (0.601–5.157)	0.303
**T stage before neoadjuvant therapy**	2.180 (0.789–6.073)	0.136
**N stage before neoadjuvant therapy**	1.646 (0.370–7.328)	0.513
**Clinical stage before neoadjuvant therapy**	1.897 (0.687–5.239)	0.217
**Chemotherapy regimen**	1.580 (0.846–2.953)	0.152
**Treatment circle**	1.398 (0.539–3.627)	0.491
**PD-L1 status**	0.651 (0.336–1.264)	0.205
**TMB**^**a**^	0.543 (0.124–2.382)	0.419
**BRCA mutation status**	0.658 (0.382–1.132)	0.130

^a^TMB, tumor mutational burden

### Biomarker analyses

Tissue samples were obtained from 16 of the 63 patients and underwent NGS examination. The median TMB was 4 mut/Mb (range 1–20.16 mut/Mb, average = 5.95 mut/Mb). Six patients (37.5%) fell into the high TMB category (TMB-H, ≥ 5.0 mut/Mb), and 10 patients (63.5%) were classified as low TMB (TMB-L, < 5.0 mut/Mb), as in other studies [20] (Supplementary Fig. 3a). Kaplan‒Meier DFS did not differ significantly between these two TMB categories (HR = 2.24 95% CI 0.60–8.28; *P* = 0.23). The median DFS was 20.7 months (95% CI 20.1 months-not reached) for TMB-H and 10.7 months (95% CI 10.2 months -not reached) for TMB-L (Supplementary Fig. 3b), respectively. The above data suggest that patients with higher TMB may tend to prolong survival and lower recurrence risk.

### Safety

No treatment-related death was observed. In Table 3, a large majority of AEs were of grade 1–2 severity, and fatigue (42.7%), neutropenia (30.0%) and diarrhea (30.0%) were the most common AEs. Grade 3–4 AEs were reported in 16.7% of patients, with leukopenia (8.3%), elevated GGT (8.3%) and asthenia (5.0%) occurring most often. This study also observed rare side effects related to immunotherapy, including xeropthalmia (1.7%), conjunctivitis (1.7%), interstitial pneumonitis (3.3%), and capillary hemangioma (1.7%).

**Table 3:** Treatment-related adverse events in all patients (*N* = 63)

AE^a^	All grade N (%)	Grade 1–2 N (%)	Grade 3–4 N (%)
**All adverse events**			
Nausea	38 (63.3%)	36 (60%)	2 (3.3%)
Vomiting	15 (25.0%)	13 (21.7%)	2 (3.3%)
Asthenia	14 (23.3%)	11 (18.3%)	3 (5.0%)
Fatigue	25 (42.7%)	23 (38.3%)	2 (3.3%)
Diarrhea	18 (30.0%)	17 (16.7%)	1 (1.7%)
Constipation	10 (16.7%)	10 (16.7%)	0 (0.0%)
Anemia	6 (10.0%)	6 (10.0%)	0 (0.0%)
Leukopenia	13 (21.7%)	8 (13.3%)	5 (8.3%)
Neutropenia	18 (30.0%)	15 (25.0%)	3 (5.0%)
Thrombocytopenia	1 (1.7%)	1 (1.7%)	0 (0.0%)
Hypoproteinemia	2 (3.3%)	2 (3.3%)	0 (0.0%)
AST increased^b^	4 (6.7%)	3 (5.0%)	1 (1.7%)
ALT increased^c^	3 (5.0%)	2 (3.3%)	1 (1.7%)
GGT increased^d^	12 (20.0%)	7 (11.7%)	5 (8.3%)
Proteinuria	2 (3.3%)	2 (3.3%)	0 (0.0%)
Arthralgia	8 (13.3%)	8 (13.3%)	0 (0.0%)
Myalgia	10 (16.7%)	9 (15.0%)	1 (1.7%)
Hand-foot syndrome	0 (0.0%)	0 (0.0%)	0 (0.0%)
Pruritus	2 (3.3%)	2 (3.3%)	0 (0.0%)
Peripheral neuropathy	6 (10.0%)	6 (10.0%)	0 (0.0%)
Gingival hemorrhage	1 (1.7%)	1 (1.7%)	0 (0.0%)
capillary hemangioma	1 (1.7%)	1 (1.7%)	0 (0.0%)
Rash	3 (5.0%)	3 (5.0%)	0 (0.0%)
Hypothyroidism	2 (3.3%)	1 (1.7%)	1 (1.7%)
Hyperthyroidism	0 (0.0%)	0 (0.0%)	0 (0.0%)
Xerophthalmia	1 (1.7%)	1 (1.7%)	0 (0.0%)
Conjunctivitis	1 (1.7%)	0 (0.0%)	1 (1.7%)
Peeling	1 (1.7%)	1 (1.7%)	0 (0.0%)
Oedema	1 (1.7%)	1 (1.7%)	0 (0.0%)
interstitial pneumonitis	2 (3.3%)	2 (3.3%)	0 (0.0%)

^a^*AE* Adverse events

^b^*AST* Aspartate transaminase

^c^*ALT* Alanine aminotransferase

^d^*GGT* Gamma-glutamyl transferase

## Discussion

Until now, there has been no multicenter real-world evidence to assess the effectiveness and safety of neoadjuvant checkpoint inhibitors combined with chemotherapy in treating early or locally advanced TNBC, which is a supplement to clinical trials [12]. The pCR rate in the current study was 34.9%, and the ORR was 82.5%, with an acceptable safety profile. During the median follow-up of 12.7 months (range 3.9 to 36.0 months), 15 of 63 patients (23.8%) had DFS events.

Neoadjuvant therapy for patients with TNBC has been revolutionized with the introduction of new agents, including anti-PD-1/L1 antibodies [12]. It is expected that most clinical studies have shown increased pCR rates or survival outcomes [11–15]; however, owing to the strict inclusion and exclusion criteria in clinical trials, real-world research is needed as a supplement [17].

Based on diverse types of research, the results of the survival analyses need to be treated with caution. As we known, the proportion of patients with more advanced tumor burden at diagnosis can impact on prognosis of neoadjuvant therapies in breast cancer. The baseline tumor burden of the patients included in this study was heavier than that in other perspective studies (41.3% of patients had > 5 cm tumors, and 71.4% had positive lymph nodes at diagnosis). For instance, in IMpassion031, approximately 76% of patients were diagnosed with stage II disease, 70% of patients had tumors ≤ 5 cm and 66% of patients had negative lymph nodes [15]. Similarly, in KEYNOTE-522, approximately 75.3% of patients were diagnosed with stage II disease, 74% of patients had tumors ≤ 5 cm and 48.3% of patients had negative lymph nodes [12]. However, the proportions of patients diagnosed with stage III and with > 5 cm tumors in our study were higher than those in above studies, which were 28.6% and 41.3%, respectively. In addition, in our study, only 28.6% of the patients had negative lymph nodes, while 71.4% had positive lymph nodes. Different from the fact that almost all people followed the prescribed regimens in prior clinical trials, only 60.3% of patients have completed eight cycles of neoadjuvant therapy in our study. Moreover, the most of check point inhibitors and chemotherapy backbone used in this real-word study were different from prior clinical trials of neoadjuvant immunotherapy in combination with chemotherapy in treating TNBC, and only 4 (6.3%) patients received a KEYNOTE-522-like regimen. Thus, the pCR rate of 34.9% in our study was lower than that of previous trials, which may be due to the differences in disease stage, proportion of positive lymph nodes, number of completed treatment cycles and distinct anti-PD-1 antibodies and chemotherapy backbone.

Of interest, one of our secondary endpoints, pCR2, defined as ypT0/Tis, which is more tolerant than the pCR regarded in most clinical studies as eradication of breast and lymph nodes. Moreover, most patients in the current study were treated with camrelizumab, and it is a novel monoclonal antibody that targets PD-1, differing from the ICI drugs such as pembrolizumab (used in KN-522), and atezolizumab (used in Impassion031) [12, 13]. Half of the patients in the current study underwent anthracycline and paclitaxel-based chemotherapy with or without carboplatin, and other patients used a carboplatin-containing regimen or eribulin, while most RCTs designed immunotherapy combined with anthracycline, paclitaxel, or carboplatin, even though direct numerical comparison may not be appropriate. Researchers found that pCR rate remained independent of PD-L1 status in both KEYNOTE-522 and IMpassion031 [12, 15]. Similarly, our study showed that the relationship between pCR rate and PD-L1 status was irrelevant as well. In addition, we observed that the rate of DFS events at 13 months in the current study was higher when compared to previous reports [12, 13, 15]. This may be explained by high proportions of T3 and N-positive patients, the high proportions of non-pembrolizumab anti-PD-1 antibody used, and some percentage of choice of non-standard chemotherapy backbone in this real-world study.

The mechanism of action and pharmacological differences in chemotherapy drugs may increase the possibility of toxicities [21]. In this study, all patients had good tolerance to the neoadjuvant treatment, and no patients died of adverse events. We observed that the application of camrelizumab was tolerable, with 60.3% of patients completing all eight cycles of treatment. There was no deterioration in physical ability when camrelizumab was added to chemotherapy. The most common AEs included nausea, fatigue, blood cell suppression and hepatic laboratory abnormalities occurring in 40% or more patients [18]. Only one patient in our study experienced a unique AE named capillary hemangioma caused by camrelizumab [22]. The immune-related AEs in this study were grade 1 or 2 and were clinically manageable. The most frequent AEs (neutropenia and elevated transaminase) were consistent with the toxicity profiles of other studies [12]. The incidences of these AEs were higher in patients who received carboplatin-containing regimen [23].

Among the 32 patients who received a germline BRCA testing in our study, only five patients were identified as having deleterious mutations. A small clinical sample size prevented a statistically significant comparison between gBRCA1/2 mutation and pCR rate [24]. Despite that, there was no significant difference in our exploratory biomarker analyses. High TMB may be associated with longer DFS, which merits further large-scale real-world studies to confirm its clinical value [23].

Although this study is the first multicenter real-world research to assess the efficacy, safety and potential biomarkers of neoadjuvant checkpoint inhibitors combined with chemotherapy in treating early or locally advanced TNBC [25, 26], it was limited by its small sample size and short follow-up period. Moreover, as a real-world study, we had no control cohort. In addition, it should be emphasized that backbone chemotherapy regimens are highly heterogeneous in this study, and may not reflect the real-world data [27].

In summary, this multicenter real-world study suggested that neoadjuvant checkpoint blockade in combination with chemotherapy may achieve a meaningful pCR rate and DFS, especially for patients with high-risk TNBC, with manageable adverse events. However, the pCR rate in this real-world study was slightly lower than those in clinical trials. Further large real-world studies investigating neoadjuvant immunotherapies in TNBC with longer follow-up are needed.

## Supplementary Information

**Additional file 1: Fig. S1.** Kaplan-Meier plot for DFS in patients treated with neoadjuvant immunotherapy.

**Additional file 2: Fig. S2.** Kaplan-Meier plot for DFS in subgroup of patients treated with anthracycline and taxane-based chemotherapy.

**Additional file 3: Figure S3.** Analysis of DFS associated with TMB.

## Data Availability

The datasets used and analyzed in the current study are available from the corresponding authors on reasonable request.
