# Supplementary material for: Neoadjuvant checkpoint blockade in combination with Chemotherapy in patients with tripe-negative breast cancer: exploratory analysis of real-world, multicenter data
Source: BMC Cancer. 2023 Jan 7;23:29. doi: 10.1186/s12885-023-10515-z (PMC9826585; doi:10.1186/s12885-023-10515-z)
Supplement: Supplementary file 1 — Additional file 1: Fig. S1. Kaplan-Meier plot for DFS in patients treated with neoadjuvant immunotherapy. [file 12885_2023_10515_MOESM1_ESM.pdf]

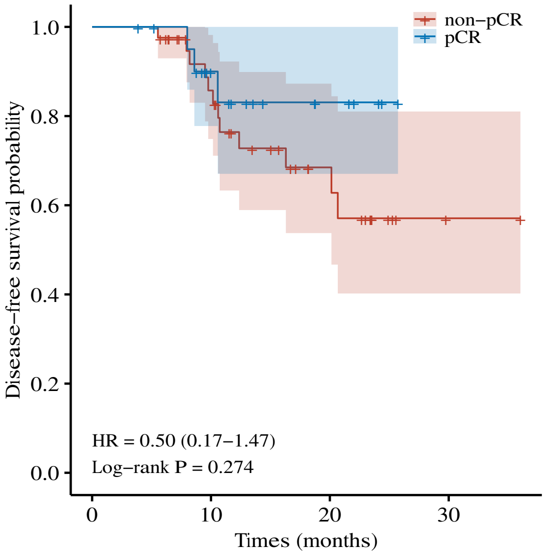

Fig S1. Kaplan-Meier plot for DFS in patients treated with neoadjuvant immunotherapy. a. Kaplan-Meier plot for DFS in all patients (n=63). b. Kaplan-Meier plot for DFS in patients with pCR1 (n=22) or non-pCR1 (n=41). pCR1 defined as ypT0/Tis and ypN0. DFS, disease-free survival. CI, confidence interval. HR, hazard ratio. pCR, complete pathological response rate.
