# Supplementary material for: Neoadjuvant checkpoint blockade in combination with Chemotherapy in patients with tripe-negative breast cancer: exploratory analysis of real-world, multicenter data
Source: BMC Cancer. 2023 Jan 7;23:29. doi: 10.1186/s12885-023-10515-z (PMC9826585; doi:10.1186/s12885-023-10515-z)
Supplement: Supplementary file 2 — Additional file 2: Fig. S2. Kaplan-Meier plot for DFS in subgroup of patients treated with anthracycline and taxane-based chemotherapy. [file 12885_2023_10515_MOESM2_ESM.pdf]

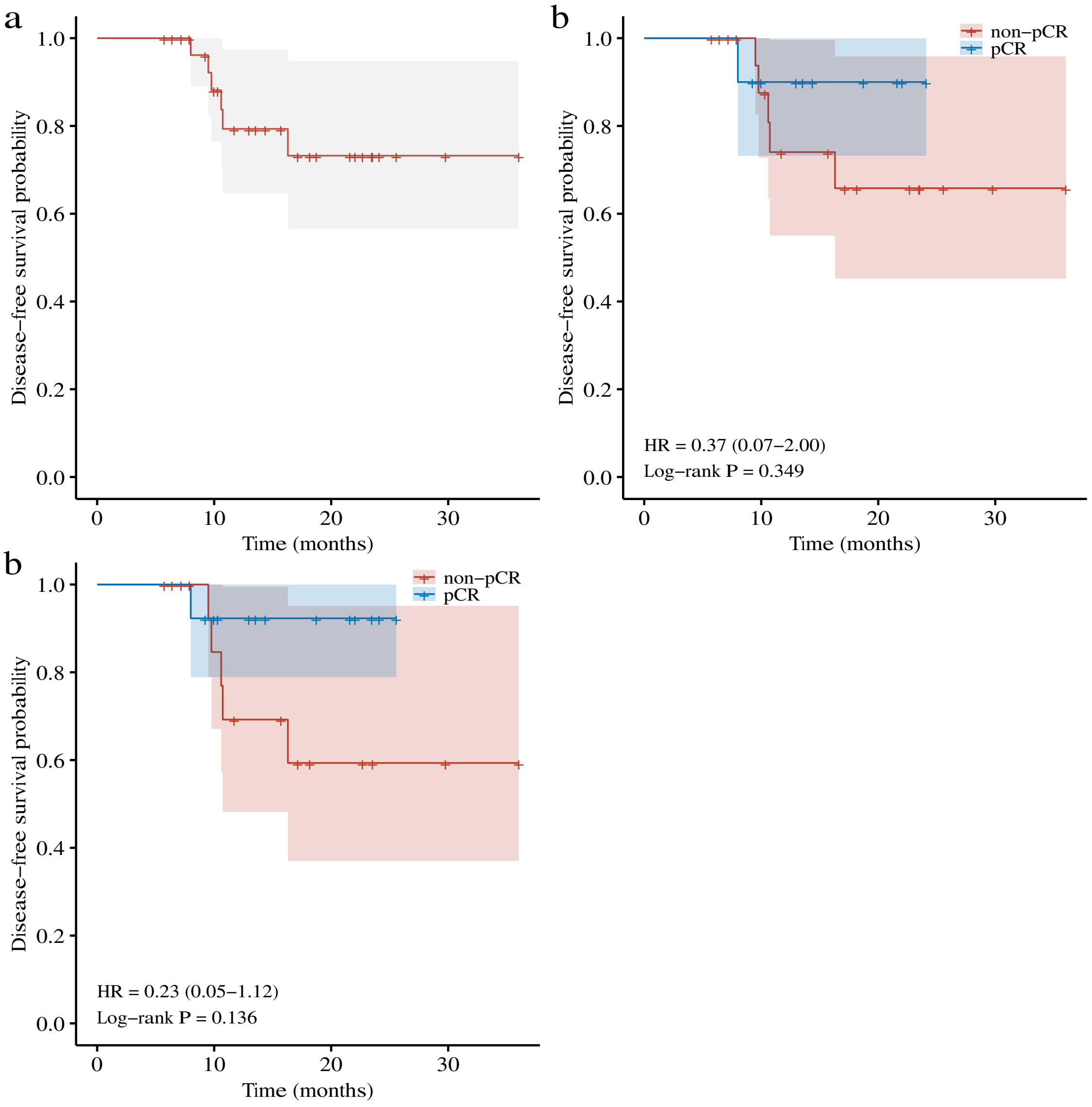

Fig S2. Kaplan-Meier plot for DFS in subgroup of patients treated with anthracycline and taxane-based chemotherapy. a. Kaplan-Meier plot for DFS in all patients (n=30). b. Kaplan-Meier plot for DFS in patients with pCR1 (n=10) or non-pCR1 (n=20). pCR1 defined as ypT0/Tis and ypN0. c. Kaplan-Meier plot for DFS in patients with pCR2 (n=13) or non-pCR2 (n=17). pCR2 defined as ypT0/Tis. DFS, disease-free survival. CI, confidence interval. HR, hazard ratio. pCR, complete pathological response rate.
