# Supplementary material for: Neoadjuvant checkpoint blockade in combination with Chemotherapy in patients with tripe-negative breast cancer: exploratory analysis of real-world, multicenter data
Source: BMC Cancer. 2023 Jan 7;23:29. doi: 10.1186/s12885-023-10515-z (PMC9826585; doi:10.1186/s12885-023-10515-z)
Supplement: Supplementary file 3 — Additional file 3: Figure S3. Analysis of DFS associated with TMB. [file 12885_2023_10515_MOESM3_ESM.pdf]

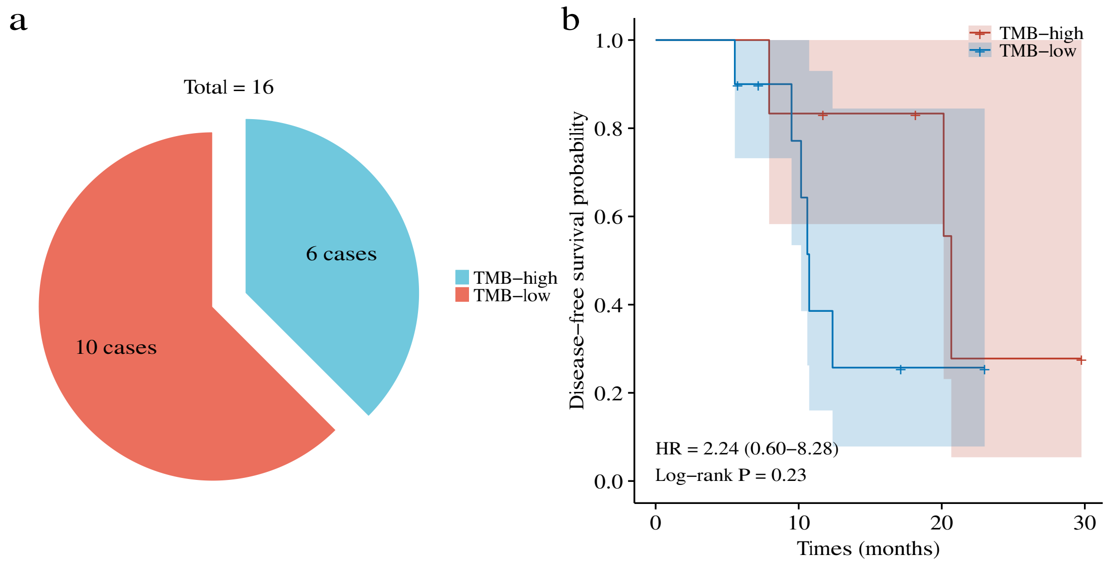

Figure S3. Analysis of DFS associated with TMB. a. Classification of patients into TMB-high (≥5.0 mut/Mb), and TMB-low groups (<5.0 mut/Mb). b. Kaplan-Meier curves for DFS according to TMB status. DFS, disease-free survival. CI, confidence interval. HR, hazard ratio. TMB, tumor mutational burden.
